# Evaluation of infliximab/tocilizumab versus tocilizumab among COVID-19 patients with cytokine storm syndrome

**DOI:** 10.1038/s41598-023-33484-6

**Published:** 2023-04-20

**Authors:** Neven Mohamed Sarhan, Ahmed Essam Abou Warda, Haytham Soliman Ghareeb Ibrahim, Mona Farag Schaalan, Shaimaa Mohamed Fathy

**Affiliations:** 1 Clinical Pharmacy Department, Faculty of Pharmacy, Misr International University, Cairo, Egypt; 2 Clinical Pharmacy Department, Faculty of Pharmacy, October 6 University, Giza, 12585 Egypt; 3 Cardiology Department, Faculty of Medicine, El-Fayoum University, El-Fayoum, Egypt

**Keywords:** Viral infection, Biomarkers

## Abstract

Coronavirus Disease 2019 (COVID-19) continues to spread rapidly. Monoclonal antibodies as well as anti-tumor necrosis factor are considered promising treatments for COVID-19. A prospective cohort study in which patients are divided into three groups. Group 1: moderate and severe COVID-19 patients received standard treatment; Group 2: moderate and severe COVID-19 patients received tocilizumab; Group 3: moderate and severe COVID-19 patients received treatment with infliximab and tocilizumab. 153 patients were recruited in the study. 40 received standard treatment alone, 70 received tocilizumab with standard treatment, and 43 received tocilizumab/infliximab with standard treatment. There was a significant difference in length of hospital stay (10.3, 8.9, and 7.6 days respectively *P* = 0.03), need for a non-invasive mechanical ventilator (4, 5, and one patient; *P* = 1.2E−8), intensive care admission (32, 45, and 16 patients; *P* = 2.5E−5), the occurrence of sepsis (18, 12, and 10 patients; *P* = 0.005) and in death (42.5%, 14.2%, and 7%; *P* = 0.0008) which were significantly lower in tocilizumab/infliximab group compared to tocilizumab and standard of care groups. Our study showed that tocilizumab/ infliximab in addition to standard of care was considered a promising treatment for moderate and severe COVID-19 patients.

Trial registration number: ClinicalTrials.gov NCT04734678; date of registration: 02/02/2021.

## Introduction

From the beginning of the COVID-19 pandemic, confirmed cases exceeded 25 million worldwide; around 100 thousand are in Egypt. Until now, several therapeutic approaches have been found for COVID-19, including antivirals, antibodies, anti-inflammatory drugs, targeted immunomodulatory therapies, and anticoagulants. However, responses to such treatments are variable among different patient populations and different stages and manifestations of the disease^1^. In mid-December-2021, COVID-19 deaths exceeded 5.3 million people globally including more than 21 thousand deaths in Egypt^2^.

Tocilizumab is a monoclonal antibody that acts as an antagonist on the interleukin-6 (IL-6) receptor. It has been approved as a treatment for idiopathic arthritis and rheumatoid arthritis^3^ and lately among the therapeutic choices for cytokine storm syndrome (CSS)^4^, which results from activation of the immune system and release of the pro-inflammatory mediators, chemokines, and cytokines^5^. The majority of COVID-19 hospitalized patients with evidence of respiratory failure show features that coincide with cytokine storm^6,7^, so it was assumed that tocilizumab as a hyperinflammatory inflammatory state could be a promising therapeutic option for these patients with CSS^8^.

Recently, Anti-tumor Necrosis Factor (TNF) therapy has been debated for its potential protective action in severe COVID-19 treatment as shown in a study by Neurath^9^. TNF may exacerbate lymphopenia by TNF/TNFR1 signalling in T cells^8^ and T cell dysfunction^10^. Therefore, TNF inhibitors could be a promising treatment for severe COVID-19 patients. A recent study confirms this finding by showing that patients with inflammatory bowel disease (IBD) who were treated with anti-TNF had significantly fewer hospitalizations and deaths compared to patients treated with other anti-inflammatory medications^11^.

In this study, we compared the outcomes of hospitalized patients with moderate to severe COVID-19 treated with tocilizumab plus standard management to those treated with infliximab/tocilizumab plus standard treatment and patients treated with standard care alone.

## Results

### Baseline patients’ characteristics

A total of 153 hospitalized COVID-19 patients were enrolled in the study. Of these, 70 patients received treatment with tocilizumab, 43 received treatment with infliximab/tocilizumab, and 40 received the standard treatment. Table 1 summarizes the baseline characteristics of the three groups. There was no statistically significant difference between the three groups when it came to mean age, inflammatory mediators (Lactate dehydrogenase (LDH), serum ferritin, C-reactive protein (CRP), and liver and heart enzymes), or any other measured parameters. However, there was a significant difference in both oxygen saturation and respiratory rate between the tocilizumab and tocilizumab/ infliximab groups compared to the standard of care group (*P* value = 0.006 and 0.004, respectively).

**Table 1:** Demographic characteristics of studied groups at baseline.

Variable as (mean ± SD)	Group 1 (Standard care) n = 40	Group 2 (Tocilizumab) n = 70	Group 3 (Tocilizumab/infliximab) n = 43	Significance *P* < 0.05
Age	59.6 ± 13.5	60.3 ± 11.3	60.1 ± 12.9	*P* = 0.258
Gender (M/F) (%)	68/32	59/41	61/39	*P* = 0.38
Oxygen saturation	89.8 ± 10.5	82.5 ± 13.1	80.8 ± 13.9	*P* = 0.006*
*P*/*F* ratio	252.4 ± 128.3	170.4 ± 82.3	164.5 ± 83.5	*P* = 0.163
Respiratory rate	20.4 ± 5.4	28.1 ± 5.3	26.9 ± 4.9	*P* = 0.004*
Maximum temperature	38 ± 0.84	38.2 ± 0.8	38.2 ± 0.94	*P* = 0.277
Baseline C-reactive protein (CRP)	230 ± 10.5	78 ± 1.1	110 ± 1.5	*P* = 0.306
Baseline D-dimmer	731.2 ± 64.4	993.9 ± 99.7	880.2 ± 96.6	*P* = 0.667
Baseline interleukin-6	12.4 ± 2.9	16.1 ± 1.7	14.8 ± 2.5	*P* = 0.14
Baseline ferritin	1017 ± 8.7	1224 ± 17.6	1267 ± 14.1	*P* = 0.337
Serum creatinine	1.8 ± 1.4	1.4 ± 0.5	1.8 ± 2.2	*P* = 0.647
Total leucocyte count (TLC)	7.9 ± 4.4	7.1 ± 3.5	8.9 ± 3.4	*P* = 0.568
Absolute lymphocytic count (ALC)	15.5 ± 9.7	15.2 ± 8.4	12.1 ± 4.7	*P* = 0.871
Neutrophils lymphocytic ratio (NLR)	8.1 ± 1.8	9.6 ± 1.1	9.2 ± 2.5	*P* = 0.122
Alanine transaminase (ALT)	51.5 ± 5.6	50.7 ± 8.4	41.6 ± 12.9	*P* = 0.233
Aspartate transaminase (AST)	51.1 ± 4.7	52.3 ± 8.3	45.2 ± 9.7	*P* = 0.802
Creatinine kinase MB (CKMB)	7.3 ± 1.6	4.6 ± 0.01	5.8 ± 1.6	*P* = 0.613
Troponin	0.01 ± 0.01	0.01 ± 3.4	0.66 ± 2.1	*P* = 0.617
Remdesivir (%)	38%	28%	34%	*P* = 0.03*
Lopinavir/ritonavir (%)	25%	9.5%	65.5%	*P* = 0.007*
Hydroxychloroquine (%)	50%	27%	23%	*P* = 0.62
Ivermectin (%)	42%	19%	39%	*P* = 0.41

*SD* standard deviation, *n* number of cases within the group, (%): *P* > 0.05.

*Significant difference < 0.05.

### Change in monitoring parameters before and after each treatment protocol

No discernible change in parameters was seen between the two sets of monitors used before and after therapy among the studied groups except for neutrophils lymphocytic ratio (NLR), which was significantly increased among group 3 compared to the other groups (*P* = 0.007), as shown in Table 2. Additionally, after treatment, CRP was significantly higher in both groups 1 and 3 compared to group 2 which showed a significant reduction in CRP (*P* = 0.004). However, for the absolute lymphocytic count (ALC), groups 1 and 3 showed a significant reduction in ALC values compared to group 2 (*P* = 0.04), as shown in Table 3.

**Table 2:** Comparison of the studied groups for the change in the monitoring parameters before and after each treatment protocol among moderate and severe COVID-19 patients.

Variable	Group 1 (Standard care) n = 40	Group 2 (Tocilizumab) n = 70	Group 3 (Tocilizumab/infliximab) n = 43	Significance *P* < 0.05
C-reactive protein (CRP)	− 62.5 ± 83.4	− 102.5 ± 94	− 83.3 ± 110.2	*P* = 0.355
Lactate dehydrogenase (LDH)	− 182 ± 344.6	− 120.5 ± 402.2	− 14.3 ± 336.1	*P* = 0.457
D-dimmer	− 1.53 ± 10.4	0.68 ± 5.4	0.46 ± 3.3	*P* = 0.567
Ferritin	− 333.2 ± 475.7	− 43.3 ± 773.7	− 29.5 ± 553.3	*P* = 0.491
Total leucocytic count (TLC)	1.73 ± 4.5	3.5 ± 5.1	2.29 ± 5.2	*P* = 0.594
Absolute lymphocytic count (ALC)	− 5.3 ± 10.8	− 2.5 ± 14.8	− 1.02 ± 5.5	*P* = 0.349
Neutrophils lymphocytic ratio (NLR)	− 0.13 ± 9.2	2.33 ± 12.1	4.9 ± 11.4	*P* = 0.007*
Alanine transaminase (ALT)	1.43 ± 7.5	33.6 ± 13.5	76.8 ± 123.5	*P* = 0.118
Aspartate transaminase (AST)	− 8.8 ± 6.5	0.73 ± 47.8	51.3 ± 58.7	*P* = 0.215
Troponin	− 0.001 ± 0.006	0.002 ± 0.008	0.66 ± 2.1	*P* = 0.587

*SD* standard deviation, variables are represented as a mean ± SD.

*Significant difference < 0.05.

**Table 3:** Comparison between the studied groups in monitoring parameters post treatment among moderate and severe COVID-19 patients.

Variable as (mean ± SD)	Group 1 (standard care) n = 40	Group 2 (tocilizumab) n = 70	Group 3 (tocilizumab/infliximab) n = 43	Significance *P* value
Time to improvement	6.3 ± 4.1	5.9 ± 3.9	6.4 ± 4.8	*P* = 0.097
Length of hospital stay	10.3	8.9	7.6	*P* = 0.03*
PF	228.7 ± 124.6	180.7 ± 62.4	210.8 ± 78.8	*P* = 0.661
C-reactive protein (CRP)	43.1 ± 7.5	46.8 ± 7.5	23.4 ± 8.2	*P* = 0.004
Lactate dehydrogenase (LDH)	297.2 ± 122.8	435.6 ± 56.4	457.1 ± 177.5	*P* = 0.110
D-dimmer	1.7 ± 1.03	2.15 ± 7.3	1.67 ± 3.2	*P* = 0.185
Ferritin	561.7 ± 27.1	1126.2 ± 86.4	937.9 ± 46.8	*P* = 0.358
Total leucocytic count (TLC)	10.4 ± 5.2	10.8 ± 5.4	11.2 ± 4.9	*P* = 0.862
Absolute lymphocytic count (ALC)	11.7 ± 5.9	13.6 ± 12.7	10.9 ± 10.8	*P* = 0.04*
Neutrophils lymphocytic ratio (NLR)	8.9 ± 1.6	14.5 ± 1.8	10.1 ± 1.5	*P* = 0.355
Alanine transaminase (ALT)	71.4 ± 7.7	102.2 ± 24.7	140.7 ± 27	*P* = 0.280
Aspartate transaminase (AST)	56.2 ± 6.9	59.2 ± 4.6	112.1 ± 6.8	*P* = 0.432
Troponin	0.02 ± 0.014	0.003 ± 0.004	0.07 ± 0.012	*P* = 0.527

*SD* standard deviation, *n* number of cases within the group.

*Significant, level of significance < 0.05.

### Change in monitoring parameters between tocilizumab and tocilizumab/infliximab groups

There was a significant difference in length of hospital stay and in monitoring parameters before and after treatment in favour of the tocilizumab/infliximab group, including CRP, LDH, ALC, and NLR which were significantly lower in the tocilizumab/infliximab group, compared to tocilizumab group. On the other hand, post-treatment liver enzymes were significantly higher in the tocilizumab/infliximab group, as shown in Table 4.

**Table 4:** Comparison between the tocilizumab and infliximab/tocilizumab groups among patients with severe COVID-19 symptoms in monitoring parameters post-treatment (Subgroup analysis).

Variable as (mean ± SD)	Tocilizumab n = 29	Tocilizumab/infliximab n = 34	Significance *P* value
Time to improvement	7.35 ± 4.7	7.13 ± 4.1	*P* = 0.147
Length of hospital stay	8.61	6.85	*P* = 0.04*
PF	210.8 ± 78.8	204 ± 97.2	*P* = 0.381
C-reactive protein (CRP)	47.8 ± 8.2	20.6 ± 6.2	*P* = 0.004*
Lactate dehydrogenase (LDH)	457.1 ± 177.5	259.7 ± 105.2	*P* = 0.0.005*
D-dimmer	1.67 ± 3.2	1.44 ± 4.6	*P* = 0.306
Ferritin	937.9 ± 46.8	790.4 ± 78.9	*P* = 0.399
Total leucocytic count (TLC)	11.2 ± 4.9	10.6 ± 5.3	*P* = 0.07
Absolute lymphocytic count (ALC)	12.2 ± 10.8	7.3 ± 12.7	*P* = 0.004*
Neutrophils lymphocytic ratio (NLR)	14.5 ± 1.5	10.1 ± 1.7	*P* = 0.01*
Alanine transaminase (ALT)	83.6 ± 9.9	140.7 ± 8.3	*P* = 0.003*
Aspartate transaminase (AST)	57.4 ± 6.2	112.2 ± 6.8	*P* = 0.0001*
Troponin	0.01 ± 0.01	0.07 ± 0.01	*P* = 0.27

*SD* standard deviation, *n* number of cases within the group.

*Significant, level of significance < 0.05.

### Clinical outcomes

There was a significant difference among the three groups in need for oxygen, mechanical ventilator, ICU admission, and development of sepsis, as illustrated in Table 5. By the completion of treatment, 34 patients in the tocilizumab/infliximab group (group 3) compared to 26 patients in the tocilizumab (group 2), and 29 patients in the standard treatment group (group 1) were severe (*P* = 4.6E−6). Eight patients in group 3, compared to 27 patients in group 2 and 18 in group 1 needed low oxygen (*P* = 0.027). Thirty patients in group 3 required high oxygen or NIMV compared to 10 patients in group 2 and 19 patients in group 1 (*P* = 0.046). The need for invasive mechanical ventilation (MV) was 1 patient in group 3 versus 5 patients in group 2 and 4 patients in group 1 (*P* = 1.2E−8). Sixteen patients in group 3, compared to 45 patients in group 2 and 32 in group 1 required ICU admission (*P* = 2.5E−5). Ten patients in group 3, compared to 12 in group 2 and 18 in group 1 showed subsequent bacterial infection, often established as sepsis (*P* = 0.005). Additionally, 3 patients died in group 3 compared to 10 patients in group 2 and 17 patients in group 1 (*P* = 0.0008).

**Table 5:** Comparison between the studied groups for moderate and severe COVID-19 patients ‘clinical outcomes.

Variable %	Group 1 (standard care) n = 40			Group 2 (tocilizumab) n = 70		Group 3 (tocilizumab/infliximab) n = 43		Significance *P* value
Severity at enrollment								χ^2^ = 23.8
Moderate/severe	27.5/72.5			62.8/37.2		20.1/ 79.9		*P* = 4.6E−6*
Need for low oxygen								
No/yes	55/45			61.4/38.6		81.4/18.6		χ^2^ = 7.26 *P* = 0.027*
Need for NIMV OR High oxygen								
No/yes	52.5/47.5			85.7/14.3		30.2/69.8		χ^2^ = 36.4 *P* = 0.046*
Need for invasive MV								
No/yes	90/10			93/7		97.5/2.5		χ^2^ = 9 *P* = 1.2E−8*
ICU admission								
No/yes	18.6/81.4			35/65		62.8/37.2		χ^2^ = 21.2 *P* = 2.5E−5*
Clinical improvement								
No/yes	22.5/77.5			14.2/85.8		20.9/79.1		χ^2^ = 4.4 *P* = 0.36
Death								
No/yes	57.5/42.5			85.8/14.2		93/7		χ^2^ = 1.4 *P* = 0.0008*
Occurrence of sepsis								
No/yes	55.8/44.2			82.9/17.1		77.5/22.5		χ^2^ = 10.5 *P* = 0.005*
Occurrence of myocarditis								
No/yes	85/15			91.4/8.6		90.7/9.3		χ^2^ = 1.2 *P* = 0.54
Occurrence of MI								
No/yes	100/0			98.6/1.4		93/7		χ^2^ = 4.7 *P* = 0.09
Occurrence of HF								χ^2^ = 1.1
No/yes	100/0			97.1/2.9		97.7/2.3		*P* = 0.57
Occurrence of PE								
No/yes	95/5			98.6/1.4		95.3/4.7		χ^2^ = 1.4 *P* = 0.49
Occurrence of hypertension								
No/yes	100/0			98.6/1.4				χ^2^ = 1.2 *P* = 0.55
Occurrence of tachycardia								
No/yes	95/5			97.1/2.9		100/0		χ^2^ = 2.1 *P* = 0.36

%: percentages of cases within the group, χ^2^: Chi-square value, S: significant difference < 0.05.

However, there was no significant difference between the three groups in the occurrence of myocarditis, myocardial infarction (MI), heart failure, pulmonary embolism (PE), hypertension, and tachycardia, as demonstrated in Table 5.

### Risk factors associated with COVID-19 severity by binary logistic regression analysis

The binary logistic regression analysis revealed that the severity of COVID-19 at enrollment and less clinical improvement were associated with the need for high oxygen and NIMV (OR = 6.45, 2.96–14.02, *P* = 1.09E−6), ICU admission (OR = 4.7, 2–11.1, *P* = 2.2E−4) and occurrence of secondary infection (OR = 3.36, 1.56–7.23, *P* = 0.002), as shown in Table 6. In addition, therapeutic interventions (tocilizumab and tocilizumab/infliximab) in group 2 and group 3 significantly reduced COVID-19 severity compared to group 1 (OR = 0.78 and 0.67, 0.64–0.91 and 0.59–0.98, *P* = 0.033 and 0.04, respectively) (Table 6). However, after correction for covariates with *P* value < 0.2 in multiple logistic regression, only need for high oxygen/NIMV (Adjusted OR = 4.98, 2.23–10.53, *P* = 0.0006), ICU admission (Adjusted OR = 3.37, 1.92–8.74, *P* = 0.001), the occurrence of secondary bacterial infection (Adjusted OR = 2.99, 1.38–6.13, *P* = 0.009) and therapeutic intervention, for both group 2 and group 3 (Adjusted OR = 0.82 and 0.73, 0.69–0.98 and 0.64–0.88, *P* = 0.041 and 0.047, respectively) remain significant as shown in Table 7.

**Table 6:** Risk factors associated with COVID-19 Severity by binary logistic regression analysis.

Risk factor	Odd ratio	95% CI	*P* value
Tocilizumab treatment (Group 2/Group 1)	0.78	0.64–0.91	0.033*
Tocilizumab/infliximab treatment (Group 3/Group 1)	0.67	0.59–0.98	0.04*
Severity at enrollment	3.78	1.66–8.62	0.001*
Need for low oxygen	0.33	0.14–0.78	0.013*
Need for high oxygen/NIMV	6.45	2.96–14.02	1.09E−6*
Need for invasive MV	1.71	0.42–3.07	0.81
ICU admission	4.7	2–11.1	2.2E−4*
HTN	0.87	0.43–1.76	0.72
DM	0.77	0.37–1.58	0.59
HF	0.719	0.65–0.79	0.94
CKD	1.3	0.31–5.45	0.71
Chronic liver disease	0.71	0.64–0.79	0.58
Ischemic heart disease	0.43	0.14–1.35	0.22
Atrial fibrillation	0.70	0.64–0.78	0.19
COPD	0.85	0.09–8.4	0.98
Asthma	0.30	0.04–2.5	0.45
Clinical improvement	0.79	0.33–1.9	0.64
Death	1.27	0.52–3.1	0.64
Occurrence of secondary infection	3.36	1.56–7.23	0.002*
Occurrence of myocarditis	0.84	0.26–2.76	0.82
Occurrence of MI	8.18	0.83–80.89	0.06
Occurrence of HF	1.29	0.11–14.56	0.99
Occurrence of PE	1.74	0.28–10.79	0.62
Occurrence of tachycardia	0.71	0.65–0.79	0.58
Occurrence of HTN	0.71	0.65–0.79	0.62

Group 1 was coded as 1; group 2 coded as 2 and group 3 coded as 3.

*Significant, level of significance < 0.05.

**Table 7:** Multiple logistic regression analysis showing risk factors associated significantly with COVID-19 Severity.

Risk factor	Adjusted odd ratio	Adjusted 95% CI	*P* value
Tocilizumab treatment (Group 2/Group 1)	0.82	0.69–0.98	0.041*
Tocilizumab/infliximab treatment (Group 3/Group 1)	0.73	0.64–0.88	0.047*
Severity at enrollment	2.64	1.45–5.82	0.003*
Need for high oxygen/NIMV	4.98	2.23–10.53	0.0006*
ICU admission	3.73	1.92–8.74	0.001*
Occurrence of secondary infection	2.99	1.38–6.13	0.009*

*Significant difference < 0.05.

### Survival analysis

**Figure 1:**
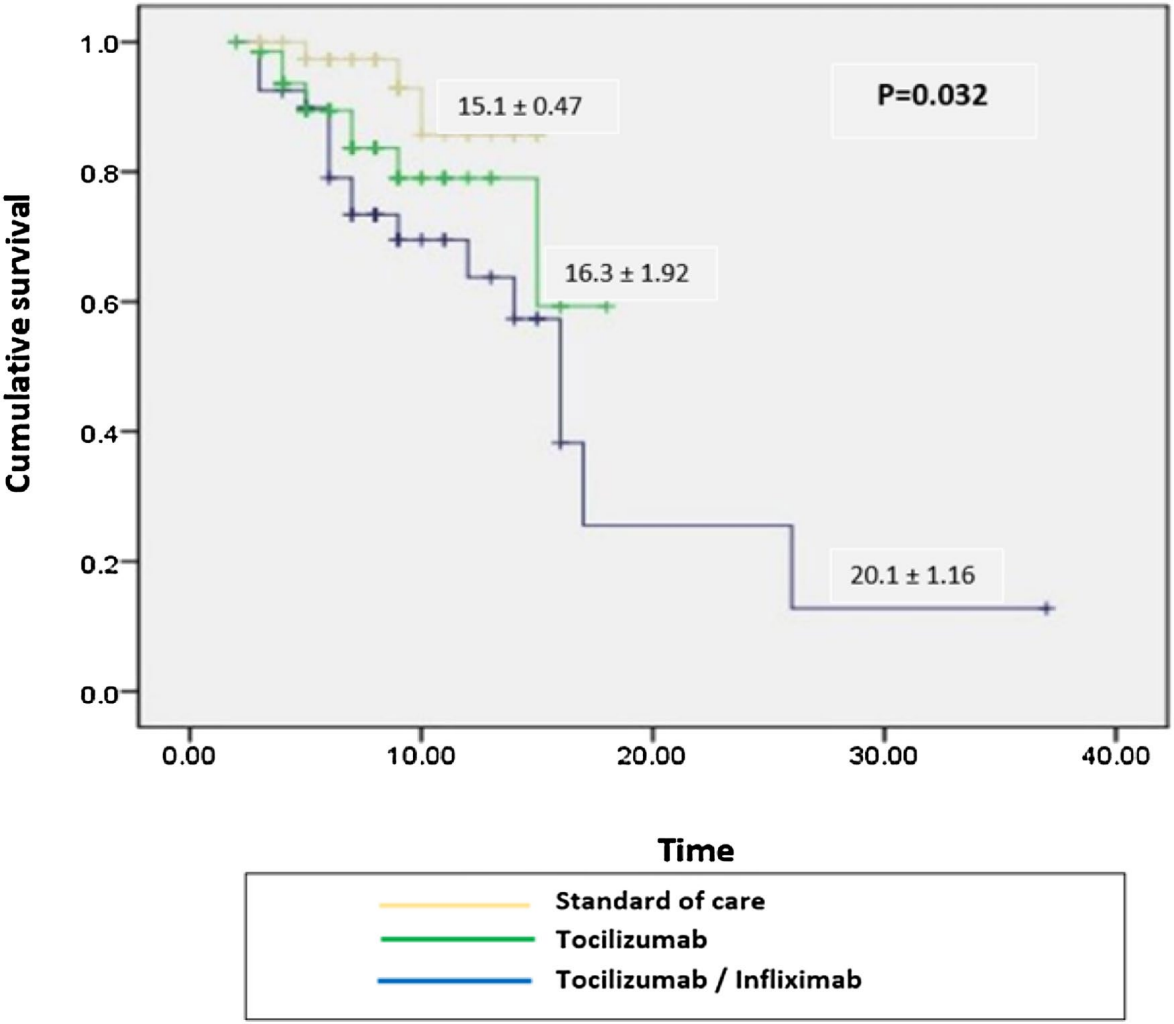
Kaplan Meier survival curve of COVID-19 patients who received Infliximab/Tocilizumab versus Tocilizumab and standard of care (*P* = 0.032).

We followed patients from admission till discharge, and overall cumulative death were reported. We found that patients who received Infliximab/tocilizumab treatment showed better survival than those who received tocilizumab and standard of care alone (*P* = 0.032), as shown in Fig. 1.

## Discussion

Covid-19 pneumonia therapeutic approaches are needed for the different races and ethnicities who were excessively affected by the pandemic^12,13^.

Covid-19 might be associated with a hyper-inflammatory state, which may play a role in the development of acute respiratory distress syndrome^14,15^. High levels of the inflammatory cytokine interleukin-6 (IL-6) were associated with severe manifestations of the Covid-19 virus, while low IL-6 levels were associated with mild disease^16,17^. Additionally, the elevation of interleukin-6 levels has been shown as a predictor of the need for ventilator support^18^.

This is the first study we are aware of to directly compare the effects of treatment with tocilizumab/infliximab versus treatment with tocilizumab in moderate-severe COVID patients.

This study showed that the need for oxygen, mechanical ventilator, ICU admission, death and occurrence of sepsis were lower significantly in the tocilizumab/infliximab group and tocilizumab group compared to the standard of care group. However, there was no significant difference between the three groups in the occurrence of myocarditis, myocardial infarction (MI), heart failure, pulmonary embolism (PE), hypertension and tachycardia. To add, the tocilizumab/infliximab group had significantly lower CRP, LDH, ALC, and NLR levels before and after therapy compared to the tocilizumab group.TNF is a pro-inflammatory cytokine that contributes to the hyperinflammatory response. TNF is increased in COVID-19 patients, and high baseline levels may be a predictor of mortality. Inhibiting tumour necrosis factor (TNF) is an example of an immunomodulatory strategy that shows significant potential as a treatment for COVID-19^19,20^. Additionally, TNF inhibitors are capable of reducing inflammation, especially pro-inflammatory cytokines associated with poor COVID patient outcomes^21^. Neuharth’s study questioned whether or not TNF inhibitors provide protection against severe COVID-19^9^.

Additionally, the TNF inhibitor, tocilizumab has been approved as one of the treatment options available for multiple inflammatory diseases^22–24^ and in several previous studies, had shown to improve COVID-19 patients with respiratory symptoms in different populations globally^25^. Nevertheless, tocilizumab clinical studies showed conflicting results among patients with COVID-19 who have varying levels of disease severity and variable standards of care approaches^26,27^.

Similar to this study findings was the Evaluating Minority Patients with Actemra study (EMPACTA), which investigated the use of tocilizumab in Covid-19 pneumonia patients who were not on mechanical ventilation. In this study, Tocilizumab lowered the incidence of the composite outcome of mechanical ventilation or mortality in hospitalized patients with Covid-19 pneumonia who were not receiving mechanical ventilators, but it did not enhance survival^28^.

Supportive of the presented study results, Stallmach et al.^29^ retrospectively explored the effect of infliximab on patients in severe conditions who have tested positive for COVID-19 compared to patients with COVID-19 who were receiving supportive therapy only. Among patients treated with infliximab, the inflammatory markers; IL-6, CRP, and LDH have shown a rapid reduction in their levels in addition to a marked increase in the lymphocytic count from baseline to post-treatment as well as an obvious clinical improvement^29^.

Contrariwise, the Surveillance Epidemiology of Coronavirus Under Research Exclusion for Inflammatory Bowel Disease (SECURE-IBD) study which compared anti-TNF administration to placebo, showed no significant association between TNF inhibitor use and the following clinical outcomes; ICU admission, ventilator use, and/or death^30^. However, infliximab use was strongly associated with a reduction in hospitalization and mortality rate consistent with the other studies^31,32^. Additionally, a case series study showed that patients who received infliximab treatment did not require a mechanical ventilator and showed no mortality compared to patients on other COVID-19 medications^33^.

In alignment with the current study, concomitant serious infection especially sepsis was significantly lower in patients on long-term treatment with infliximab (Li, 2020). However, in studies evaluating infliximab use as a treatment for the septic shock of bacterial origin, patients showed no significant difference in mortality rate though infective and inflammatory markers did not deteriorate^34,35^.

Similar to the current study, Hachem et al. showed a rapid resolution of lymphopenia for patients with baseline lymphopenia. These patients, upon discharge had a significant increase in lymphocytic and monocyte counts from baseline and a significant reduction of the inflammatory mediators involved in the pathogenesis of severe COVID-19 infection^36^. Additionally, patients on infliximab therapy showed improvement in respiratory parameters in terms of SpO2/FiO2 and reduced need for ventilator support^36^.

Indicators of the severity of illness, such as the necessity for intensive care, multi-organ failure, and mortality, have been linked to elevated serum concentrations of tumour necrosis factor alpha (TNF) and its established regulatory targets, such as interleukin-6 (IL-6) and ferritin^6,37^.

In the current study, there was a decrease in ferritin and CRP in the tocilizumab/infliximab group. In the context of these results, Liu et al. concluded that patients with elevated IL-6 levels at baseline (> 10 pg/ml) were positively correlated with increased baseline levels of CRP, LDH, ferritin, and D‐dimer^38^. Additionally, this study showed that patients who received Infliximab/Tocilizumab had significantly better survival compared to the standard of care, and the parameter neutrophil lymphocytic ratio (NLR) was suitable to distinguish between those patients who could eventually be discharged and those who died with AUC of 76.5%^38^.

Similar to this study findings was Salama et al.’s^28^ study, in which there was reduced mortality among severe COVID-19 hospitalized patients who received tocilizumab added to standard treatment versus those treated with placebo. In contrast to our study results, two previous studies suggested that IL-6 receptor inhibition has an extensive therapeutic effect on patients with Covid-19. On the contrary, the results of a limited number of unpublished randomized controlled trials are not suggestive of its use^39,40^. The mean time to hospital discharge was 2.11 days shorter in the Infliximab/Tocilizumab group than in the Tocilizumab and standard of care group. Similar to our study findings was Salama et al.^28^ study, which showed that tocilizumab plus standard care showed a significantly shorter hospital stay by 1.5 days than placebo plus standard care.

## Conclusion

Our study showed that infliximab/tocilizumab added to standard treatment was more effective than tocilizumab added to standard treatment in reducing death and biochemical inflammatory markers and in improving clinical outcomes among severely hospitalized patients with Covid-19.

## Methods

### Patients and setting

From December 2020 through June 2021, a cohort of COVID-19 hospitalized patients in the inflammatory phase were recruited for a prospective observational study at Teacher's Hospital, Cairo, Egypt, ClinicalTrials.gov (NCT04734678). Written informed consent was obtained from all patients recruited in the study.

#### Study design

The present investigation is a prospective cohort study in which patients are divided into three groups. Group 1 includes hospitalized COVID-19 patients who received standard treatment. Group 2 includes hospitalized COVID-19 patients who received treatment with tocilizumab PLUS standard management. Group 3 includes hospitalized COVID-19 patients who received treatment with infliximab/tocilizumab PLUS standard management.

#### Eligibility criteria

Patients over 18 who were hospitalized with pneumonia confirmed by chest CT scan and tested positive for COVID-19 infection using RT-PCR were included in the study. We included patients who had CSS which was detected by inflammatory markers elevation; either C-reactive protein (CRP) ≥ 100 mg/L or ferritin ≥ 900 ng/mL, along with lactate dehydrogenase (LDH) > 220 U/L and interleukin-6 level (IL-6) > 10 pg/ml. In addition to one of the following: respiratory rate ≥ 30 respirations/min, oxygen saturation ≤ 93%, ratio of pressure arterial oxygen partial pressure to inspired oxygen fraction (PaO2/FiO2) < 300 or who showed worsening of pulmonary areas of consolidation, defined as increase in number and size of patches^41,42^.

Patients with evidence of concurrent bacterial infection, use of other immunosuppressant, and levels of alanine aminotransferase (ALT) or aspartate aminotransferase (AST) that are five times higher than the upper range of normal were excluded. Additionally, patients who received treatment with anti-TNFα in the previous month and show hypersensitivity to any TNFα inhibitor as well as active or latent tuberculosis were excluded. Patients who received any COVID-19 vaccine or those who were previously infected with SARS-COV were also excluded from the study.

#### Treatment

All patients received the standard treatment of 400 mg of hydroxychloroquine once daily, or 400/100 mg of lopinavir/ritonavir twice daily, or remdesivir 200 mg as a loading dose followed by 100 mg once daily as a maintenance dosage, in addition to dexamethasone 6 mg once daily for 7–10 days. Anticoagulant enoxaparin is administered subcutaneously once daily as a preventative measure if the D-dimer is between 500 and 1000, and twice daily as a therapeutic measure if the D-dimer is greater than 1000. Patients also received supportive treatments of quetiapine (25 mg once a day at bedtime) and paracetamol (1 g every 6 h). Intravenously (IV) tocilizumab was administrated at a dose of 4–8 mg/kg/day divided into two doses, 12–24 h apart when steroid therapy has failed to improve the condition for 24 h. After 24 h, a second dose was administered if the patient experienced any sign of respiratory worsening, including the requirement of ventilator support invasive or non-invasive. Infliximab was given as an IV infusion at a dose of 400 mg only once. The cardiologist was the one responsible for detecting any cardiovascular complications.

### Study outcomes

#### Primary outcomes

The length of patients' hospital stays, and admission to the intensive care unit (ICU) were first evaluated as primary outcomes.

#### Secondary outcomes

Secondary outcomes include death, the use of NIV or invasive mechanical ventilation, the development of secondary infections (such as bacterial or fungal infections), and an elevation in liver enzymes above three times the normal threshold.

### Sample size calculation

By using a two-sided hypothesis and an alpha of 0.05, we found that a sample size of 134 patients gave us over 80% power to detect an effect size of f2 of 0.16. With the knowledge that some of our recruits would inevitably drop out, we made sure to get in an extra 10%.

### Statistical analysis

The data were analyzed using SPSS, a statistical package for the social sciences, version 22.0 (SPSS, Chicago, IL-USA). The mean and standard deviation are presented for continuous data. Numerical and percentage-based displays of categorical information are used. The normality of the data was investigated with the help of the Kolmogorov–Smirnov test and the Shapiro–Wilk test. When comparing two groups based on numerical variables that followed a normal distribution, we used the student’s *t* test. The analysis of variance (ANOVA) was used to compare the groups and to track the evolution of the data over time, while the Chi-square test was used to evaluate the continuous variables. The Mann–Whitney U test was utilized to make group comparisons for non-normally distributed variables. Over time, we compared two numerical variables using the Wilcoxon signed-rank test. When comparing the two groups in terms of categorical information, Fisher's exact tests were used. Biochemical and clinical predictors of each outcome of interest were evaluated using regression analysis. In addition to the variables that were already considered in the univariate analysis because of their association with the outcome (clinical covariates), we also considered additional variables with *P* values of less than 0.2. All clinical predictors in the final model had to have a *P* value of 0.05 or lower to be considered significant. Survival curves were calculated using Kaplan–Meier and compared with a log-rank test. All *P* values are two-tailed, and a value of 0.05 was taken to indicate statistical significance.

### Ethical approval

The study protocol was reviewed and approved by the institutional review boards (IRBs) of faculty of Pharmacy, October 6 University (PRC-Ph-2206019). Written informed consent was obtained from all patients recruited in the study. This study was performed according to the Declaration of Helsinki.

## Data Availability

The datasets generated during and/or analyzed during the current study and the study protocol are available from the corresponding author upon reasonable request.
